# The “Motor of the Day”: Parent and School-Age Children’s Cognitions, Barriers, and Supports for Breakfast

**DOI:** 10.3390/ijerph16183238

**Published:** 2019-09-04

**Authors:** Kaitlyn M. Eck, Colleen L. Delaney, Rashel L. Clark, Miriam P. Leary, Karla Pagan Shelnutt, Melissa D. Olfert, Carol Byrd-Bredbenner

**Affiliations:** 1 Department of Nutritional Sciences, Rutgers University, 26 Nichol Avenue, New Brunswick, NJ 08901, USA; 2 Division of Animal and Nutritional Sciences, West Virginia University, 1194 Evansdale Dr. G28, West Virginia University, Morgantown, WV 26506, USA; 3 Department of Family, Youth, and Community Sciences, University of Florida, Gainesville, FL 32611, USA

**Keywords:** Children, Parents, Breakfast, Social Cognitive Theory, Focus Groups

## Abstract

Despite the many benefits of regular breakfast consumption few parents and children consume this meal every day and research examining the determinants of breakfast consumption is limited. Thus, the purpose of this study was to examine breakfast-related cognitions (i.e., beliefs, attitudes, facilitators, barriers) of parents and school-age children (ages 6–11 years) using the constructs of Social Cognitive Theory as a guide. Parents (*n* = 37) and children (*n* = 41) participated in focus group discussions held in community settings in 3 states (FL, NJ, WV). Data were qualitatively content analyzed to detect trends. Parents felt breakfast was important for numerous reasons. Parents expressed concern about the healthfulness of some traditional breakfast options, preferring breakfasts containing mostly fruits, vegetables, and protein and fewer carbohydrates. Parents identified lack of time as the greatest barrier to breakfast consumption. To overcome this barrier, they utilized grab-and-go foods, prepared breakfast ahead of time, and got up earlier. Utilizing the school breakfast program was another strategy mentioned, however some were concerned about the nutritional quality of this option. Children recognized the importance of breakfast and cited several benefits. The greatest barrier to breakfast identified by children was feeling rushed in the morning. To overcome barriers, children suggested having a morning routine, selecting or preparing breakfast foods ahead, and relying on parents to encourage breakfast consumption. The effectiveness of interventions aiming to improve breakfast intake may be improved by addressing parent and child breakfast-related cognitions and tailoring interventions to address their needs.

## 1. Introduction

Childhood obesity continues to be a public health concern as nearly one in five school-aged children (i.e., 6 to 11 years old) in the United States are obese and rates of obesity increase with age [1,2]. Childhood overweight and obesity track into adulthood, with 55% of obese children becoming obese adolescents and 80% of obese adolescents becoming obese adults [3]. The negative health consequences associated with childhood obesity are numerous and include early onset of chronic diseases, such as diabetes and hypertension [4,5]. Many overweight and obese children also experience weight teasing, which can have negative psychological consequences [6].

Regular breakfast consumption has repeatedly been shown to be associated with healthy body weights [7,8] and children who skip breakfast are more likely to be overweight than children who consume breakfast regularly [9,10,11,12]. Individuals who skip breakfast also consume less healthful diets than regular breakfast consumers [9,13,14]. For instance, children who skip breakfast obtain a greater percentage of their daily energy intake from snack foods and consume fewer fruits, whole grains, and dairy foods, which are often staples at the breakfast table [14]. In contrast, breakfast eaters have higher intakes of many nutrients, including vitamin D, folate, calcium, and fiber [14,15,16].

The foods selected for the breakfast meal determine its nutritional benefits. For example, individuals who eat breakfast cereals consume less sodium and more sugar, carbohydrates, and fiber than individuals who have non-cereal breakfasts [15], but they also consume fewer added sugars overall than breakfast skippers [17]. Those whose breakfast meals include animal proteins (e.g., eggs, meat, poultry, fish) consume more saturated fat and sodium daily than breakfast skippers [18]. 

Children who regularly eat breakfast perform better academically than children who skip breakfast [19,20]. Children who consume a nutritious breakfast score higher in literacy tests than those who skip breakfast [21]. The association between breakfast intake and improved school performance is particularly notable in under nourished children [21]. Some researchers hypothesize that improved academic performance may result from children’s increased ability to concentrate when they are adequately fed [22,23]. Others note that implementation of school breakfast programs (the setting for many studies on the effects of breakfast on school performance) improve school attendance, an important contributor to overall improved school performance [21].

Despite the numerous benefits breakfast consumption offers, many youth do not consume breakfast regularly. It is estimated that 10 to 34% of children and adolescents regularly skip breakfast [24]. National Health and Nutrition Examination data indicate that breakfast consumption rates decline in U.S. children as they move from early elementary school to high school, with 4% of 4 to 8 years olds, 14% of 9 to 13 years olds, and 20% of 14 to 18 years olds not consuming their first meal of the day until after 11 a.m. [25]. 

Interventions aiming to improve breakfast consumption rates may benefit from applying constructs from the Social Cognitive Theory (SCT). SCT is a behavior change theory that highlights the effects of both personal and environmental factors on behaviors [26,27,28]. The central theme of this theory is reciprocal determinism, which describes the simultaneous and reciprocal influence of an individual’s behavior on his or her environment and the environments effect on behavior [26,27,28]. As an example, a parent (or the family as a whole) can alter aspects of their home environment to facilitate the performance of a behavior, such as eating breakfast more regularly. The concept of reciprocal determinism makes SCT an ideal theoretical framework for guiding the development of interventions targeting dietary behavior change. Additional constructs of SCT, collective efficacy (confidence in the group’s [e.g., family’s] ability to perform a behavior) and observational learning (learning by watching others, such as a child learning from behaviors modeled by a parent or older sibling) support the use of SCT in family-based behavior change interventions [26,27,28]. SCT also posits that behaviors are influenced by perceived barriers and facilitators to performing a behavior and by outcome expectations (beliefs about the potential benefits and detriments of the behavior), suggesting the importance of understanding these determinants of behavior and their importance in behavior change interventions [26,27,28].

Regular breakfast consumption confers an array of health benefits, yet for many, intake is erratic and/or comprised of less healthy foods. There is a need for interventions to improve the frequency of breakfast intake. The most effective interventions are predicated on a clear understanding of the factors associated with behavioral determinants [26,29], yet limited research has sought to elucidate determinants of eating breakfast, i.e., the underlying cognitions (i.e., beliefs, attitudes), barriers, and facilitators of breakfast. Most qualitative studies related to breakfast consumption have focused on school breakfast and breakfast behaviors of adolescents [30,31,32], with little attention given to younger children and breakfasts eaten at home. Additionally, breakfast research rarely has been predicated on behavior change theoretical constructs that could identify factors that could motivate and facilitate adoption of healthier breakfast practices. Therefore, this qualitative research study undertook an examination of these factors in parents and school-age children (6 to 11 years) with the goal of informing the creation and tailoring of health promotion materials grounded in the behavior change constructs of the SCT [26,27,29] that aim to improve breakfast consumption patterns in these audiences.

## 2. Materials and Methods

This qualitative, focus group study was approved by the Institutional Review Boards for Protection of Human Subjects at the authors’ universities. Parents gave informed consent for themselves and their children. Children also gave verbal assent prior to participating. Focus groups were conducted during summer and fall, 2017.

### 2.1. Sample

Parents who had at least one school-age child (between the ages of 6 and 11 years), made most of the decisions about what foods to purchase and serve in the home, lived in one of three geographic regions within in the United States (West Virginia, New Jersey, or Florida), and spoke either English or Spanish were invited to take part in a 60-min focus group addressing small, easy changes they could make to their homes and lifestyles to help kids grow up even healthier. To minimize participation bias, recruitment notices made no mention of diet, nutrition, healthy eating, or breakfast. Participant recruitment notices in both English and Spanish were posted electronically (websites, email listservs) and flyers were distributed at locations frequented by parents (e.g., workplaces, schools, community centers). Parents received a stipend of $25 for completing the focus group. School-age kids (ages 6 to 11 years) were recruited to participate in 30-min focus group discussions using electronic and printed messages targeted to parents. In exchange for participation, children were compensated $15. 

### 2.2. Instrument

Before the focus group discussion commenced, parents received a short paper questionnaire gathering demographic information (e.g., age, highest education level, number and ages of children, frequency of breakfast consumption). Child participants provided similar information. The focus group guides were semi-structured and developed and administered by trained researchers using standard procedures [33,34]. The focus group guide was developed to elicit parent and child attitudes and beliefs related to breakfast and to identify factors influencing breakfast consumption. The SCT provided the structure for the focus groups, which endeavored to elucidate attitudes toward eating breakfast, barriers to eating breakfast, strategies for overcoming barriers to breakfast intake, and confidence in the ability to eat breakfast on a regular basis (Table 1) [26,28]. The content of the focus group guide was reviewed by a panel of experts for congruence with the study purpose and SCT, pilot-tested, and refined [35]. 

All researchers completed training and practice sessions to maintain uniformity in data collection across researchers and data collection sites. Focus group discussions were led in the primary language of the parents (i.e., English or Spanish). Children’s focus groups were led in English because all were fluent in English, including Latino children. Focus groups with younger children (ages 6 to 9) were conducted separately from those with older children (ages 9 to 11). Researchers aimed to keep focus group size small to allow for rich contributions from each parent or child involved. Comprehensive notes of focus group discussions were taken by a second trained researcher who transcribed them within 48 h of concluding the focus group. The researcher who led the focus group reviewed the notes for clarity, comprehensiveness, and accuracy. Spanish language focus group notes were translated into English by the researchers leading and recording notes of the focus groups. Both researchers independently reviewed the notes then met to reach agreement on their contents.

### 2.3. Data Analysis

The Statistical Package for the Social Sciences (SPSS) version 21.0 (IBM Corp, Chicago, IL, USA) was used to generate descriptive statistics summarizing questionnaire data. Content analysis was conducted independently by three researchers trained in qualitative data analysis to identify trends in the focus group data [36,37]. Standard content analysis procedures yield impartial, methodical descriptions [38] that allow researchers to extract “replicable and valid inferences from the data to their context” [39]. (p. 21). Researchers discussed their independent content analysis findings and reached a common agreement. Throughout data collection, data were constantly analyzed and compared to ascertain when the point of data saturation (or information redundancy) was reached and data collection should conclude [36,40].

This qualitative study used a quote replete reporting method to present results. This method was employed because the presentation of qualitative data “rests on verbal expressions” [41] Throughout the content analysis process, researchers identified quotes from parents and children to include as data points supporting interpretation of raw data [42]. Another reason the quote replete reporting method was used relates directly to a goal of this study, that is to yield results that could inform the development of intervention materials aiming to improve breakfast consumption. Replete reporting of quotes provides insight into how parents and children verbalize thoughts related to breakfast and gives researchers and practitioners the data required to tailor breakfast intervention materials on the target audiences’ linguistic patterns. Tailored communications promote central processing by the target audience, which enhances the likelihood of stimulating behavior change [43]. However, few nutrition communications are able to be tailored to audience verbal expressions because of the tendency of researchers to summarize findings with scant use of quotes. 

## 3. Results

A total of 37 parents participated in 1 of 11 focus group discussions about breakfast. Most parents were female (95%) and had at least some college education (78%). The parents’ average age was 38.97 ± 5.38 standard deviation (SD) years and they had 2.73 ± 1.31 SD children under the age of 18 living in their homes. Fewer parents participated in a Spanish language focus group compared to those participating in an English language focus group (35% and 65%, respectively). Geographic distribution of parents was fairly even across states (*n* = 14 FL, *n* = 11 NJ, *n* = 12 WV), with an average of 3 parents per group (range: 2 to 6). 

A total of 41 children participated in 1 of 13 focus group discussions about breakfast. About half of the children were girls (51%). Children’s average age was 8.56 ± 1.80 SD years. Children reported having 2.17 ± 3.81 SD older and 1.27 ± 1.48 SD younger siblings. Geographic distribution of children was fairly even across states (*n* = 15 FL, *n* = 14 NJ, *n* = 12 WV) with an average of 3 children per group (range: 2 to 5). Children and parent participants were not related, except for one parent and child. 

### 3.1. Parent Focus Groups 

Results from a brief questionnaire preceding the focus groups indicated that parents ate breakfast an average of 5.82 ± 1.56 SD days/week. Breakfast frequency was similar across parents’ primary language spoken and geographic location. In addition, qualitative focus group data did not vary by language or geographic location.

#### 3.1.1. Parents’ Breakfast Attitudes and Observations

Parents believe that breakfast is “*extremely important—it is the motor of the day*” because breakfast helps kids “*wake up*” to the challenges of the day and “*gives them* [kids] *energy*”, acknowledging that, “*if they don’t eat, they don’t function very well, which you can physically see*”. Parents agreed that breakfast consumption is important to performance at school because kids “*won’t be able to focus without it* [because] *they will feel hungry*”.

Parents noted that breakfast affected children’s behaviors, with one citing that research on “*child behavior and performance supports breakfast*”. When kids eat breakfast they are “*happier,* [and have] *less behavior problems in the classroom*”. Additionally, when their children miss breakfast they become “*hangry*” and are “*cranky and not cooperative*”. 

Parents felt that the quality of the breakfast was important (“*what they are eating affects it* [their behavior], *too… PopTarts^®^ aren’t going to be healthy enough to get through to lunch*”). Parents described healthy breakfast options as “*oatmeal, eggs, something with whole grain fiber, protein, and fruit*”, “*milk*”, “*good cereals—Rice Krispies^®^, multigrain Cheerios^™^, shredded wheat,* and [not] *bad cereals—Fruit Loops^®^, chocolate cereal*”. Other parents defined “healthy cereal” as “*ones with less sugar and more fiber*”. Parents reported that “*a good breakfast is protein, although my kids eat a lot of carbs—bagels, cereal, bread*” and would “*like to incorporate more fruit…and add more vegetables…while having less of the carbs*”. Others remarked that “*a good breakfast* [included] *something that will stay with them and they enjoy eating*” and that “*eating anything* [at breakfast] *is better than nothing*.”

Parents observed that as their children have gotten older, they “*eat a lot more. They want a hearty breakfast*” and that older kids have “*more of an opinion on what they want.* [Their] *taste buds have changed*”. Parents also said that older kids, “[have a] *desire to eat it* [breakfast]*, we used to prompt them more and now she asks for it…it has gotten easier*” and indicated their kids “*now are the gatekeepers to their breakfast and even control the amount they eat.* [They may have] *just a slice of bread one day and 3 waffles the next day. They might eat something different each day”*. 

Numerous parents indicated that “*during the week, they* [kids] *eat at school*”. Parents had mixed feelings about meal offerings at schools with some stating that “*They are giving them the best breakfast at school*”. One mother described school breakfast as “*not great, it’s disgusting*”. Others expressed concern about not knowing what is served at school, “*I worry a lot because I don’t know what they are feeding them at school*”, so it is “*better to have breakfast at home because I am making it, I know what is in it*”. 

#### 3.1.2. Parents’ Perceived Barriers to Breakfast

The most commonly cited barrier to breakfast was lack of time. Some parents felt that “*work impedes us*” and is a barrier to breakfast consumption (“*sometimes I can’t* [serve breakfast]*, we don’t have enough time since I work*”). Parents also described their mornings as hectic stating that, “*when you wake up late, you have to run around the house to get out the door on time.*” “*Sometimes you forget about breakfast on busy mornings because you’re trying to get lunch together* [as well]”. Parents noted that “*If we are running late, that prevents it* [breakfast]”, “*If we have something to do or somewhere to go,* [we] *don’t have time to make breakfast*” and that “*Sometimes there isn’t time to eat breakfast*”. A few noted that kids take priority for breakfast, “*when I wake up super late, I won’t eat breakfast, but I make sure that they* [kids] *eat”*. And, “*If I’m taking too long in the morning to make them breakfast, they will even figure out what to eat themselves*.” Parents also indicated that television was “*disruptive*” and contributed to the morning time crunch, “*sometimes my husband turns on TV in the morning, which slows down kids’ morning routine*” and it “*can derail our schedule.”*

Eating breakfast as a family was not common. “*I don’t eat with them, I am doing other things*.” Most parents felt that there isn’t time to sit down together for breakfast during the week, but some had more time for family breakfasts on the weekends. One commented, “*the only time we eat breakfast as a family is when I serve it for dinner.*”

Parent behaviors also can present barriers to breakfast. One parent described herself as “not a breakfast person, so it’s a new thing for me that we have to save time for them to sit down and eat”. “I don’t eat [breakfast], so my children don’t eat and we get hangry.” Others indicated they did not plan breakfasts ahead of time: “we eat whatever we have—I don’t really plan it.” 

Parents reported that children impact family breakfast habits. For example, kids may not be hungry (“*my son isn’t always hungry when he wakes up in the morning*”). “*Breakfast can be a real struggle*” if children are finicky eaters *(“my one kid doesn’t like breakfast foods, which makes it difficult*”). Additional time is required for “*negotiating with them”* to find *“something they like* [for breakfast]. Varying preferences also was a challenge because “*children know what they want* [to eat] *and it’s difficult to adapt to what each child eats*”.

#### 3.1.3. Parents’ Strategies for Overcoming Barriers to Breakfast

Parents had an array of strategies for overcoming barriers to breakfast. To cope with a lack time for breakfast, parents suggested having ‘on-the-go’ options for mornings when “*I don’t have time to make breakfast*”, such as “*granola bars*”, “*dry cereal*”, and “*fruit yogurt*”. Another suggestion was to “*have options around that are easier than always having to cook a hot breakfast*”, such as “*having boiled eggs ready*”, “*buy*[ing] *pancakes premade and pop them in the microwave*”, and “*find*[ing] *easy meals—like* [cooking an] *egg in a microwavable cup*”.

Parents also suggested getting up earlier so that there is time to eat breakfast. “We wake up early, not too early, but early enough to make sure everyone has breakfast.” “I intentionally get up early so I don’t feel time pressured. I have confidence knowing I’ve set a pattern for myself to prevent time being a factor.” However, others commented, “I can’t get up earlier and my minutes are counted” and “I don’t think anything can help unless you are morning people.”

Another strategy for overcoming time scarcity in the morning was giving children responsibility for their own breakfast (“At 6 years old, they can access most of the breakfast options themselves. They have the skills, for the most part, to prepare their own breakfast”). Another mother said, “I make sure what they like is convenient for them to prep on their own if I am not there”. Parents also get kids involved by asking kids to “think about what they want for breakfast and have them choose when grocery shopping…that way it’s there and maybe [kids will be] more willing [to eat breakfast].” 

Many acknowledged that it was helpful for kids to have breakfast at school. But when eating breakfast at home or school wasn’t possible, some parents “*send them* [kids] *with something to school*”, have kids “*eat in the car*”, or “*send dry cereal in a bag with him to eat on the bus*”. 

Parents agreed that planning ahead could alleviate time barriers to breakfast. “Starting [breakfast] preparation the night before make[s] breakfast time easier”. Noting that “time is an issue for most people”, parents “recommended getting everything ready the night before. Clothes are laid out. Lunches are packed. So, then in the morning, we just have to eat breakfast and get dressed.” Other ways parents plan ahead is by making food readily accessible (“I leave him cereal and milk before leaving to go to work”).

Parents also felt that having an established routine helped them fit breakfast into their daily lives. Routines included getting up sufficiently early to allow time for breakfast, preventing schedule disruptions by not “*turning on the TV in the morning,”* and making eating breakfast an expectation starting at an early age (“*accustoming them* [to breakfast] *from when they are babies*.”) 

Even when time is scarce, parents made it a priority to feed children breakfast: “*I always make sure that they at least have eaten something before leaving the house*”. Parents indicated school lunch schedules also were a motivator for serving their children breakfast because “*lunch is so late, so if they didn’t eat breakfast, they will be starving all day*”.

To overcome a lack of desire to eat breakfast and cope with picky eaters, parents suggested reminding kids that “*you’ve got to eat breakfast, it will make you grow healthy and strong*” and by offering a “*variety of foods*”, “*one day cereal, another oatmeal, another day bagels*”. Parents suggested offering a “*choice basket*” that contains “*options where they* [kids] *can choose what they want to eat in the morning”*. They also pointed out that breakfast “[doesn’t] *have to be traditional breakfast foods, as long as they eat breakfast*”. So, “*if they* [kids] *aren’t breakfast people, give them choices—do you want a shake, rice and beans, or a quesadilla?*” and to remember that “*It is important to try different types of foods, don’t give up so easy because they will always say no in the beginning, but might eventually try it*”. 

A few parents suggested making breakfast an enjoyable event so children want to consume this meal. “*My kids love to read the comics, so that’s what they do during breakfast*”. “*Music helps us, we don’t usually talk in the morning*”. An additional point was, “*If you know your child is not a good eater, wake them up earlier so they eat breakfast calmly*”. 

### 3.2. Children’s Focus Groups

Survey results indicated that children ate breakfast an average of 5.69 ± 2.15 SD days/week, with 79% of the children reporting they ate breakfast every day and one child reporting never eating breakfast. For the children not eating breakfast daily, intake ranged from never having this meal to eating it 6 days per week. Children’s breakfast frequency was similar across geographic locations. Focus group data were similar across child age groups.

#### 3.2.1. Children’s Breakfast Attitudes and Behaviors 

Children felt that consuming breakfast promotes good health “because it gives you energy to start the day”, “helps you get stronger and taller”, “keeps you happy and healthy”, and prevented negative behaviors and attitudes (“Eat breakfast! Makes me cranky if I don’t eat”). “If you don’t eat breakfast you will be grumpy and sad, and if you get used to not eating breakfast everyday then you’ll be grumpy and sad all the time”. Many children referred to breakfast as “the most important meal of the day”. 

Kids agreed that waking up hungry (“in the morning your belly gets hungry”) facilitates their desire to eat breakfast, noting that “My brain tells me to eat breakfast” and “In the morning, I just wake up and feel really weak without breakfast”. Children agreed that having breakfast prevents them from being hungry (“[breakfast is] really important or your stomach will growl”) and without breakfast kids felt “extra hungry when lunch comes along”. Kids also described stomachaches caused by hunger from breakfast skipping, “you feel sick—stomach hurts and you feel like you’re going to throw up, but you don’t have any food in your stomach to throw up”. 

Kids felt that a healthy breakfast should have “*a little bit of all food groups*” and include foods like *“fruits, eggs*”, “*applesauce, yogurt, cereal, and pop tarts*”. Other commonly mentioned breakfast foods included bagels, pancakes, yogurt, bananas, grits, and toast. Some children also mentioned atypical breakfast foods such as cucumbers, sandwiches, and salad. One child commented, “*I like to change what I eat every day, but it has to be healthy*”. 

Overall kids felt parents “should eat the same thing” as kids for breakfast because “healthy foods are the same for adults and kids”. Children indicated that they “usually do not eat together as a family for breakfast” because “parents cannot eat with us because they are getting ready for work”, but many said that on “Saturdays and Sundays we eat together. Big breakfast”. 

#### 3.2.2. Children’s Perceptions of their Parents’ Attitudes towards Breakfast 

Children thought that their parents feel breakfast is “*Very important. They want you to stay healthy”*. Kids reported that parents wanted them to be healthy and not feel hungry at school (“*because lunch time is too late at school. I get hungry in the morning. So, they want me to eat my breakfast*”). Kids also believed that their parents thought that breakfast is important for school performance because they “*don’t want you to fall asleep in class. They care about my learning*”. Kids also remarked that breakfast was important to parents because “*It keeps our energy for the day because they don’t want us to be tired*” and prevents kids from being “*hungry and grumpy*”. Kids knew breakfast was important to their parents because “*they will wake me up to eat breakfast*”, “*they make us eat breakfast every day*”, and “*they always make it for us every single day*”. 

#### 3.2.3. Children’s Perceived Barriers to Breakfast 

Children said that one of the key barriers to breakfast is that they are often “*still sleeping*”, “*in a rush*”, or they are “*running late to school*”. Others acknowledged that competing activities can prevent them from eating breakfast, stating that “*Time can sometimes cause me not to eat breakfast—like when I play sports. Saturdays and Sundays are the worst days*”. Kids reported that they skip breakfast because “*Sometimes I am not hungry in the morning and I like to sleep in. I wake up at the latest time possible, so I don’t have time for breakfast*”, that they “*May not like what they are having* [for breakfast]”, “*don’t feel in the mood*”, there is “*nothing to eat*”, or that “*sometimes my sister snacks before breakfast, so that stops her*”. Distractions such as TV, playing outside, and siblings (“*I distract my brother sometimes, so he doesn’t eat breakfast”*) also caused kids to miss breakfast. One kid said, “*sometimes I plan on eating breakfast at school and when I get to school, I forget to eat*”. 

#### 3.2.4. Children’s Strategies for Overcoming Barriers to Breakfast 

Children agreed that having a “*routine helps you eat breakfast*”. Kids felt that parents could “*set a timer for you so you can eat breakfast at a certain time*” to help establish a breakfast routine. Kids also thought they could take a role in ensuring they ate breakfast indicating they could “*set an early alarm and get up early to make breakfast*” or “*try and go to sleep early*”. Kids also felt parents could address time pressures in the morning by **“***as soon as you* [the parent] *wake up, make breakfast before they* [kids] *wake up and have it on the table so it’s ready when they* [kids] *wake up*” or “*go to work later*”.

Children also thought that they could “*get parents to get breakfast foods they like*” so kids would be more willing to eat breakfast. To make it easy for kids to have breakfast, children indicated that it helps when “*cereals are left out* [on the counter]” and when breakfasts are planned ahead (the day before, “*we pick out what we want to eat in the morning”; “make breakfast the night before so if you’re running late in the morning, you can put it in a container and eat it on the way out*”*).* Kids also recognized that they could help prepare breakfast, remarking that “*I get up and help my mom make it, I make eggs, I make it with my mom*” and “*maybe I can make breakfast for them* [my parents]”. Another indicated she could help her family have breakfast more often by asking parents to get “*more cereals for breakfast and having a chart with options*.”

The kids pointed out that parents could remind children about the importance of breakfast—“*tell them it starts their day, it’s healthy”*. Kids also suggested that parents could provide some grab-and-go options (“*Have something that is easy to take on the go, like a banana*”) or make foods easily accessible (“*have it on the table*”). Kids also recommended that parents “*give them choices*” of breakfast foods and that kids and parents “*try new foods together*”. Children felt that parents could make breakfast more interesting by making *“something fun, like faces with fruit on pancakes or chocolate chip pancakes*” or serving *“dessert—yogurt with blueberries and strawberries*”.

Kids reported that eating breakfast at school could prevent breakfast skipping but noted that there were some constraints. “*You need to get to school early*”. “*You can eat breakfast at school,* [but] *you have to eat it fast*”. “*If you don’t eat breakfast at home, you can eat at school, but some of us have to pay and some of us don’t*”. “*Sometimes I eat breakfast at school, but I get in trouble if I eat at school* [because family has to pay]”. 

A few children suggested that parents could “*force*”, “*trick*”, “*pay”* or reward kids with treats to get them to eat breakfast. A few thought parents should “*punish kids if they don’t eat breakfast—no snacks, take toys away*” or “*make rules and be strict*”. Other suggestions to promoting breakfast eating were to “*make us sit at the table*” and “*lock the closet full of junk food*”. One child thought that there was nothing parents can do “*to help their kids eat breakfast*”.

#### 3.2.5. Children’s Perceptions of the Influence of Family Members on Breakfast Consumption 

Children’s views on whether family members’ breakfast choices influenced their own breakfast choices were mixed. Some reported that “*parents’ breakfast intake doesn’t have much effect*” on them because “*the whole family usually eats breakfast, but at different times*” (“*I don’t see my parents eat breakfast, my mom eats after I go to school and my dad is a teacher—he eats way before I am awake*”). Other kids reported that “*if I wake up and see my parents eating, then I might think about eating, too*”. Kids also noted that they were more likely to try new things if they saw their parents eating them (“*If we see something we like that they are eating, then maybe we might want to try it the next day*”). Children felt parents could influence their children through the foods they prepare “*if they make something healthy for breakfast and it’s on the counter, we can eat it*”. Some kids saw their parents as the enforcers of healthy breakfast choices and reported that “*If they don’t eat breakfast with me, I will sneak other things like sweets into my breakfast*”. Siblings also influence intake at breakfast. One child said “*My little sister gets sugary cereal, so then we all eat the cereal she eats*”, and another said “*My brother eats what I eat*”. 

## 4. Discussion

This study aimed to identify the cognitions, barriers, and facilitators of breakfast for parents and school-age children and use them to develop recommendations for interventions, predicated on the SCT, targeting improved breakfast behaviors of families with school-age children. The recommendations are summarized in Table 2 and each is discussed below. Some of the recommendations outlined in Table 2 are currently employed by existing interventions [44,45]. This study further justifies their use and suggests additional methodology that can be used in tandem to improve the effectiveness of the intervention materials.

### 4.1. Breakfast Outcome Expectations

Study findings indicate that parents reported numerous positive outcome expectations associated with breakfast consumption, including better school performance, mood regulation, and overall functioning. The role of breakfast in weight management [46,47,48], however was not mentioned by focus group participants. This lack of awareness suggests an opportunity for future nutrition education programs to inform parents about the inverse relationship between breakfast consumption and BMI for both children and adults [7,46,47,48,49], particularly in the case of high-protein breakfasts [50]. As posited by the SCT, improving outcome expectations related to breakfast (i.e., belief that breakfast consumption has important outcomes, including weight management), could increase breakfast intake behaviors for parents and their children [26,29,51]. 

Similar to parents, children also associated numerous positive outcome expectations with breakfast consumption. They felt that eating breakfast was important for growth and development, mood regulation, and to prevent feeling hungry, but children did not mention improved school performance or enhanced concentration as a benefit of eating breakfast. Children who do not believe that consuming breakfast improves their ability to concentrate in class are significantly more likely to skip breakfast than children who believe breakfast improves concentration [52]. As noted in Table 2, future nutrition education programs can improve child outcome expectations related to breakfast consumption by emphasizing the association between breakfast intake and academic performance. 

Despite recognizing several benefits of breakfast, both parents and children most miss breakfast at least sometimes. It is possible that both parents and children overstated the importance placed on breakfast consumption as they did not feel comfortable sharing a negative opinion in a group setting. Or, it may reflect the reality of barriers families face. 

### 4.2. Overcoming Barriers to Facilitate Eating Breakfast 

Indeed, parents and kids encounter numerous barriers to eating breakfast, with the most common obstacle being lack of time, which is similar to findings from other studies [53,54]. To overcome this barrier, some parents suggested having children consume breakfast at school (Table 2). This is a viable solution for many children; however, national participation in the federally funded school breakfast program is about half the rate of participation in the school lunch program [55,56,57]. Low participation in school breakfast may be partially explained by parents’ lack of knowledge about the quality and healthfulness of breakfast provided at schools. This highlights an opportunity for schools offering breakfast to invite parents to school breakfast so they can see what is being served and learn how the school foodservice staff constructs the menu to meet children’s taste preferences while also applying federal nutrition requirements that meet children’s nutritional needs. This firsthand knowledge may increase parents’ willingness to have kids eat breakfast at school. 

Children also suggested eating breakfast at school as a strategy for facilitating eating breakfast regularly. However, as participants pointed out, barriers to eating breakfast at school also exist, including needing to arrive early, having to eat quickly due to limited time for this meal, and cost. In response to time barriers, some schools have implemented “Breakfast in the Classroom” or “Breakfast after the Bell” programs, which allow kids to eat breakfast in the classroom typically during the first period of the day [58,59]. As suggested in Table 2, continued efforts to remove barriers related to eating school breakfast, including cost, could improve school breakfast program participation rates and child health. 

### 4.3. Observational Learning to Promote Breakfast Consumption

In addition to reporting barriers to breakfast consumption, parents and children shared many unique and valuable strategies for overcoming obstacles. For example, strategies for overcoming challenges with picky eaters included offering a variety of foods, including non-traditional breakfast foods, and getting children involved in planning breakfast. To cope with time scarcity in the morning, parents suggested quick, easy breakfast options, such as microwaving eggs and having grab-and-go options on hand. Parents also had suggestions for improving the atmosphere during breakfast by playing upbeat music while preparing and eating breakfast to energize the family. Sharing these “parent-tested” strategies in nutrition education interventions could promote behavior change by providing vicarious peer modeling and facilitators to behavior change [27,29,51].

Parents were concerned about the quality of some ready-to-eat breakfast foods and the overall breakfast meals they provided for their children at home. For instance, parents’ opinions on the healthfulness of breakfast cereals were polarized as “healthy cereals” and “bad cereals”. “Bad cereals” were defined by parents as those with a “high” sugar content (i.e., fruit-flavored cereals, chocolate cereals). Other studies also have reported parent concern about the sugar content of cereals [60]. There was a general lack of awareness that the added sugar in breakfast cereals accounts for only a small percentage of added sugar in the diet, far less than the sugars contributed by baked goods, desserts, and soft drinks [61]. In addition, parents did not seem to know that there is value in adding some sugar to improve the palatability of the naturally bland flavors of grains to promote intake or that breakfast cereals are nutrient dense, which points to important consumer knowledge gaps. Helping parents understand that breakfast cereal eaters consume greater amounts of vitamins and minerals and less fat than non-cereal eaters and that research suggests that children who eat pre-sweetened breakfast cereals are not at increased risk of becoming overweight or obese [62] could increase parent confidence in the healthfulness of breakfast options they offer while also promoting breakfast consumption. Helping parents to realize that ready-to-eat breakfast cereals, even pre-sweetened ones, can be a healthy breakfast option may reduce breakfast skipping. 

Both parents and children acknowledged the influence of observational learning (e.g., children’s observations of parents’ or older siblings’ behaviors) on children’s breakfast behaviors. Children also realized that they can influence their siblings’ dietary intake. Given some of the less than ideal breakfast behaviors reported in the literature [25] and in the study current study, highlighting the importance of modeling of healthy eating by parents as well as older siblings in nutrition education programs has the potential to improve breakfast behaviors, a tenet also supported by the SCT (Table 2) [26,29].

A few parents mentioned children can get their own breakfasts, and some kids recognized that they were able to prepare their own breakfasts as well. Involving kids in food preparation and empowering them to prepare their own meals has numerous benefits, including improved diet quality, greater child self-efficacy for healthy food selection, and increased child pride [63,64,65]. As suggested in Table 2, teaching parents strategies for helping children learn to make their own breakfast (and for siblings and parents) and build their food preparation self-efficacy could improve breakfast intake now while also preparing kids for future responsibilities. In addition, gaining cooking skills during the growing years positively correlates with diet quality in adulthood [66], which underscores the importance of incorporating cooking skills in nutrition education interventions. 

### 4.4. Study Limitation and Strengths 

The majority of children in this study ate breakfast daily. National Health and Nutrition Examination data indicate, though, that breakfast consumption rates decline in U.S. children as they move from elementary school to high school [25]. Among U.S. teens, only 35% of girls and 43% of boys consume breakfast daily [67]. Thus, continued efforts to make breakfast a strong habit during the elementary school years, in hopes this behavior will track into adolescence and beyond, is an important goal for nutrition education programs.

A limitation of the study was most participants ate breakfast on most days of the week. It is possible that these reports are artificially inflated by social desirability bias, however it is important to consider that the barriers and facilitators experienced by frequent breakfast consumers maybe different than those experienced by those eating breakfast infrequently. Another limitation is that the sample size was modest and collected in only three geographic regions. However, the data generated are rich and informative and reached the data saturation point prior to terminating data collection. Additionally, the parent focus groups were conducted in two languages allowing a more diverse and inclusive sample. Further, the use of trained researchers to conduct focus groups allowed for consistent and thorough data collection across all states. Although individual interviews allow participants more time to speak, focus group size was intentionally kept small to allow all participants to have sufficient opportunities to share their thoughts. Additionally, focus groups offer the opportunity for participants to share ideas and explore similarities and differences which was suitable to achieving the study goal of developing recommendations for interventions targeting breakfast behaviors. A further strength is the use of the quote replete reporting method that will permit the development of intervention tailored to the linguistic patterns of the target audience.

### 4.5. Conclusion and Future Directions 

This is one of the first studies to qualitatively analyze both parent and school-age children’s attitudes and beliefs related to breakfast using behavior change theoretical constructs to identify factors that could facilitate adoption of healthier breakfast practices (outlined in Table 2). School-aged children are beginning to make their own health-related decisions, but are still reliant on their parents, who are the food gatekeepers in the home, to make many of these decisions. For this reason, findings from this study that elucidate the attitudes and beliefs and perceived barriers and facilitators of both groups is an important contribution to the literature that has the potential to guide the development of more effective interventions aiming to improve breakfast intake, boost overall family nutritional health, and prevent childhood obesity. Future studies and breakfast promotion programs should examine the effectiveness of incorporating the findings of this study and recommendations in Table 2 in improving frequency and healthfulness of breakfast consumption in families with school-age children.

## Figures and Tables

**Table 1:** Focus Group Discussion Questions for Parents and Children Organized by Social Cognitive Theory (SCT) Constructs.

SCT Construct	Parent Question	Child Question
Perceived importance	How important is it to you that your children to eat breakfast?	How important do you think it is for you to eat breakfast?
		How important do you think your parents feel it is to eat breakfast?
Barriers to eating breakfast	What prevents you from getting children to eat breakfast?	What stops you from eating breakfast?
Strategies (facilitators) for overcoming barriers	What helps you get children to eat breakfast?	What helps you eat breakfast often?
	What advice would you give parents to encourage them to serve their families breakfast?	What advice would you give parents to encourage them to be sure their families eat breakfast?
		What are some things you can do to help your family have breakfast more often?
Observational Learning	How do you think your breakfast eating choices affect your children’s breakfast eating?	How do you think your parents’ breakfast intake affects your breakfast intake?
		How do you affect your family’s breakfast intake?

**Table 2:** Focus Group Derived Recommendations for Future Interventions Targeting Breakfast Behaviors in Families with School-Age Children Organized by Social Cognitive Theory (SCT) Constructs.

SCT Construct	Recommendations for Future Breakfast Interventions
Outcome Expectations	Expand breakfast outcome expectations to include weight management.
Outcome Expectations	Expand breakfast outcome expectations for children to include improved school performance.
Facilitation	Invite parents to school breakfast to enhance their knowledge of the food served during school breakfast and the methods used to construct healthy, child-friendly menus.
Facilitation	Promote factors to overcome barriers to school breakfast, such as “Breakfast in the Classroom”, “Breakfast after the Bell”, and universal free breakfast.
Facilitation	Provide parents and children with ideas for expanding breakfast options beyond traditional breakfast foods.
Facilitation	Increase parents’ repertoire of quick, easy, and varied breakfast food options.
Facilitation	Help parents build breakfast planning skills.
Facilitation	Provide reminders about the importance of breakfast.
Facilitation/Observational Learning	Share “parent-tested” strategies to increase frequency of breakfast consumption.
Facilitation/Outcome Expectations	Teach parents about the nutritional qualities of breakfast foods, including ready-to -eat breakfast cereals, to help them understand the contributions these foods can make to supporting regular breakfast consumption.
Self-efficacy	Build children’s confidence in their ability to prepare and eat healthy breakfasts on a regular basis.
Observational Learning	Encourage parents and older siblings to model healthy breakfast eating behaviors for children.
Facilitation	Provide parents with strategies to help children learn to make their own breakfast.
Facilitation/Outcome expectations	Teach kids recommended child feeding strategies to prepare them for being responsible for feeding children in the future.
